# High Prevalence and Genetic Polymorphisms of *Legionella* in Natural and Man-Made Aquatic Environments in Wenzhou, China

**DOI:** 10.3390/ijerph14030222

**Published:** 2017-02-24

**Authors:** Leyi Zhang, Yi Li, Xin Wang, Zhihui Shangguan, Haijian Zhou, Yuejin Wu, Lianghuai Wang, Hongyu Ren, Yun Hu, Meifen Lin, Tian Qin

**Affiliations:** 1 Wenzhou Center for Disease Control and Prevention, Wenzhou 325000, China; zjwzliyi@126.com (Y.L.); vicky158564@126.com (Z.S.); spirit995378@126.com (Y.W.); wz2036894245@163.com (L.W.); wzcdchy@163.com (Y.H.); meizi7015@163.com (M.L.); 2 Qingjiangpu District Center for Disease Control and Prevention, Huai’an 223001, China; xinwangbiology@hotmail.com; 3 State Key Laboratory for Infectious Disease Prevention and Control, National Institute for Communicable Disease Control and Prevention, Chinese Centre for Disease Control and Prevention, Beijing 102206, China; zhj_0901@163.com (H.Z.); hongyu2690@sohu.com (H.R.); 4 Collaborative Innovation Center for Diagnosis and Treatment of Infectious Diseases, Hangzhou 310003, China

**Keywords:** pathogen monitoring, *L. pneumophila*, genetic diversity, molecular typing, public health, environmental water source

## Abstract

Natural and engineered water systems are the main sources of Legionnaires’ disease. It is essential from a public health perspective to survey water environments for the existence of *Legionella*. To analyze the main serogroups, genotypes and pathogenicity of the pathogen, a stratified sampling method was adopted to collect water samples randomly from shower water, cooling tower water, and local public hot springs in Wenzhou, China. Suspected strains were isolated from concentrated water samples. Serum agglutination assay and real-time PCR (Polymerase chain reaction) were used to identify *L. pneumophila*. Sequence-based typing (SBT) and pulsed-field gel electrophoresis (PFGE) were used to elucidate the genetic polymorphisms in the collected isolates. The intracellular growth ability of the isolates was determined through their interaction with J774 cells and plating them onto BCYE (Buffered Charcoal Yeast Extract) agar plates. Overall, 25.56% (46/180) of water samples were *Legionella*-positive; fifty-two strains were isolated and two kinds of serogroups were co-detected from six water samples from 2015 to 2016. Bacterial concentrations ranged from 20 CFU/100 mL to 10,720 CFU/100 mL. In detail, the *Legionella*-positive rates of shower water, cooling tower water and hot springs water were 15.45%, 13.33%, and 62.5%, respectively. The main serogroups were LP1 (30.69%) and LP3 (28.85%) and all strains carried the dot gene. Among them, 52 isolates and another 10 former isolates were analyzed by PFGE. Nineteen distinct patterns were observed in 52 strains isolated from 2015 to 2016 with three patterns being observed in 10 strains isolated from 2009 to 2014. Seventy-three strains containing 52 from this study and 21 former isolates were selected for SBT analysis and divided into 25 different sequence types in 4 main clonal groups belonging to 4 homomorphic types. Ten strains were chosen to show their abilities to grow and multiply in J744 cells. Taken together, our results demonstrate a high prevalence and genetic polymorphism of *Legionella* in Wenzhou’s environmental water system. The investigated environmental water sources pose a potential threat to the public where intervention could help to prevent the occurrence of Legionnaires’ disease.

## 1. Introduction 

*Legionella* widely exists in natural freshwater and artificial water sources, and belongs in the class of opportunistic pathogens [1]. The species *Legionella pneumophila*, accounts for approximately 90% of human infection with the *Legionella* genus that has 16 serogroups identified. Serogroup 1 (LP1) is the most common group implicated in Legionnaires’ disease. Other serogroups were also detected in past outbreaks with the second most common being serogroup 6 (LP6) [2,3,4]. Bemander et al. studied an outbreak of *L. pneumophila* in a Swedish hospital where they detected and isolated serogroups 4, 6, 7, and 10 [5]. The main sources of infection causing outbreaks and legionellosis epidemic are water from the air-conditioning cooling system, condensed water system, cold and hot water supply systems, hot springs, and spa baths, amongst others. Possible sources of human infection are faucets, shower heads, fountains and evaporative condensers [6].

The European working group for *Legionella* infections (EWGLI) reported a total of 243 cases of legionellosis from 2007 to 2008, with 87 of them associated to hot and cold water supply system, thirty-two of them related to cooling tower system contamination, and 6 cases connected to fountain and spa baths [7]. In Spain, *Legionella* from the hotel spa pool and domestic water supply system were responsible for the outbreak of Legionnaires’ disease from 2011 to 2012 [8]. In the US, engineered water systems such as cooling towers are known to be major sources for legionellosis and outbreaks [9]. Legionellosis had also been an increasing concern in other countries. In Poland, a study carried out from 2001 to 2008 in Warsaw revealed that the frequency of *Legionella* was 78% in the hospitals’ hot water systems, 68% in industrial plants, 93% in residential buildings, and 68% in hotels [6]. In four provinces of Gabon, Africa, 29 isolates of *Legionella* spp. were frequently found in hospitals particularly in hot water systems at a rate of 11.6% in a 2013 investigation [10]. In China, since the first case of legionellosis in 1982, the disease has been reported sporadically in many cities. However, these were rarely reported as outbreaks, which could be attributed to the lack of a nationwide monitoring network of *L. pneumophila* and its pathogenicity as well as incomplete epidemic data on legionellosis [11,12,13]. Previous cases in China mostly arose from water in the cooling tower and condensation water of air conditioners. Unfortunately, *L. pneumophila*is also prevalent in the shower, pipelines and hot springs waters.

Strain differentiation is necessary for the identification of sources of contamination and determination of routes of transmission in water distribution systems, which could in turn enable us to more accurately detect outbreaks and limit the spread of *L. pneumophila* infections. A variety of subtyping techniques have been used for epidemiological typing, including pulsed field gel electrophoresis (PFGE) and sequence-based typing (SBT). PFGE is a highly discriminative epidemiological method for subtyping *L. pneumophila* [14], and is the most commonly applied approach to investigate Legionnaires’ disease outbreaks and trace the source of infection [15]. SBT could not only meet the need for distinguishing outbreak isolates but also provide a rapid, highly discriminatory, and reproducible seven-gene molecular typing method that has now become an internationally recognized procedure for genotyping *L. pneumophila* Isolates [16,17].

In Wenzhou, located in the southeastern part of China, *L. pneumophila* monitoring of water in cooling towers has been going on for 7 years. However, the other possible sources of infection remained uninvestigated. Thus, the aim of this study was to investigate other water sources that could expose large number of people to *L. pneumophila* (e.g., hotels, hospitals and hot spring resorts). Pulsed-field gel electrophoresis (PFGE) and sequence-based typing method (SBT) were used to analyze the pathogen’s genetic characteristics. The pathogenicity was measured by intracellular growth ability of the isolates. This study is critical in guiding policies for the management of possible sources harboring *L. pneumophila*to prevent outbreaks of legionellosis.

## 2. Materials and Methods

### 2.1. Sample Collection

Water samples from various sources were collected from June 2015 to March 2016 in Wenzhou. For the collection of water from the cooling towers and showers, we randomly selected 20 hotels and 4 hospitals as collection points. In each hotel, 1 water sample from the cooling tower and 3 water samples (one sample from each room shower totaling 3 rooms) were collected. In each hospital, 2 or 3 water samples (one from each cooling tower) were collected per the number of hospital cooling towers. Ten to fifteen water samples from hospital wards were collected per the number of respiratory wards. For the collection of water from the hot springs, we collected water from four hot springs hotels situated in Wenzhou. Two samples were collected in one year, once in summer and another in winter; each time, 5 pools were randomly chosen for collecting water samples.

For all water sources, sodium thiosulfate (0.1 mol/L) was added in empty pre-sterile containers to inactivate chlorine. Five hundred milliliters of water were collected each time and is referred to as one sample. All procedures for collection and pretreatment of samples were based on the recommendations found in ISO 11731:1998. The samples were delivered to the laboratory for testing immediately after collection.

Prior study of *L. pneumophila* isolation was conducted with the same sample collection procedure. Twenty-one water samples from cooling tower of hotels and hospitals were collected from 2009 to 2014.

### 2.2. Legionella Isolation

Two hundred milliliters of water from each sample was filtered through a 0.45 μm membrane. The membrane was cut into pieces by sterile scissors followed by the addition of 5 mL of sterile dilution buffer, which was placed on a vortex bath for 2 min thereafter. After re-suspension, acid treatment was applied (ISO 11731:1998). Each sample (100 μL processed solution) was plated on GVPC agar (Oxoid Microbiology Products, Hampshire, UK). The plates were cultured in an incubator at 37 °C with 5% CO_2_ for 10 days. We examined plates and screened the colonies each day for *Legionella*. *Legionella* colonies were identified using Gram staining, the l-cysteine requirement test and serum agglutination using polyclonal antisera (Denka Seiken Co. Ltd., Tokyo, Japan), and were counted.

### 2.3. Real Time PCR Assay

The primers and TaqMan probes were designed per *L. pneumophila*’*s* 5S rRNA genes and the conservative *dot* gene [18,19,20]. The primers and probe for the 5S rRNA are as shown in Table 1.

The pre-screened colonies were reconfirmed upon the detection of these two genes of interest. Pure cultures of *L. pneumophila* were obtained as per the reference method [18] and the genomic DNA extraction was performed according to the manufacturer’s instruction (QIAamp DNA Mini Kit (QIAGEN, Venlo, The Netherlands)). The real time PCR mixture contained primers (0.2 μM for each primer), 0.4 μM probe, 100 ng bacteria DNA as templates and 10 μL PCR mixture (Premix Ex Taq, Takara Co. Ltd., Kusatsu, Japan) in a final volume of 20 μL. Amplification reactions were performed using a ViiA™ 7 Real-Time PCR System (Applied Biosystems, Thermo Fisher Scientific, Waltham, MA, USA). The thermal profile was 2 min at 95 °C (activation of the TaKaRa Ex Taq HS polymerase), followed by 15 s at 95 °C, and 1 min at 60 °C for 40 cycles.

### 2.4. Pulsed Field Gel Electrophoresis (PFGE)

We followed a standardized PFGE protocol for *L. pneumophila* subtyping as previously described [21]. Cell suspensions were prepared in polystyrene tubes with optical density of 3.8 to 4.0 measured using a Densimat photometer (BioMe’rieux, Marcyl’Etoile, France). The cell suspensions were then embedded into 1% SeaKem Gold molten agar. Thereafter, the agar was soaked in the cell lysis buffer with proteinase K (20 mg/mL) so that the embedded cells were lysed completely. *Legionella* slices were digested using AscI 20U per slice (New England Biolabs, Ipswich, MA, USA) for 4 h at 37 °C. The strain serogroup H9812 of *Salmonella braenderup* was adopted as a molecular standard. Electrophoresis was run with a switch time of 6.8 s to 54.2 s for 19 h, voltage of 6.0 V/cm, and electric field angle of 120 degrees using a CHEF-DRIII system (CHEF-Mapper^®^ XA, Bio-Rad, Hercules, CA, USA). Images were captured using a Gel Doc 2000 system (Bio-Rad, Hercules, CA, USA) and converted to TIFF files. The TIFF files were analyzed using the BioNumerics version 7.5 software (Applied Maths, Kortrijk, Belgium). Similarity analysis of the PFGE patterns was evaluated by calculating the Dice coefficients (SD) [22] and clustering was built by applying the unweighted-pair group method with average linkages (UPGMA). Sixty-two strains in total were selected for PFGE analyzation, 52 strains were isolated from the time period 2015 to 2016 by PFGE, while 10 strains were isolated and identified previously, from the time period 2009 to 2014.

### 2.5. Sequence-Based Typing

Seventy-three strains were analyzed by SBT: Fifty-two strains were isolated during 2015 to 2016 from various water sources, and 21 strains were sporadically isolated previously from 2009 to 2014 from the cooling tower in hotels and hospitals. The European Working Group for *Legionella* Infections (EWGLI) suggests seven genes (*flaA*, *pilE*, *mip*, *asd*, *mompS*, *proA* and *neuA*) for genotyping using the standard sequenced-based typing (SBT) method; the 5.0 version can be found here: http://www.hpa-bioinformatics.org.uk/legionella/legionella_sbt/php/sbt_homepage.php. The genomic DNA extraction method and the PCR procedure were according to the Sequence-Based Typing protocol for epidemiological typing of *Legionella pneumophila* version 5.0. The amplicons were sent for sequencing by Sangon Bio-technique Company (Beijing, China). The SBT database that was available on the EWGLI website (http://www.ewgli.org/) was used for nucleotide analysis, and the sequences were compared with those in the SBT database, which were also available on the website. BioNumerics software version 7.5 (Applied Maths, Brussel, Belgium) was applied to cluster analysis of related sequence types (STs), representing their genetic relationships.

### 2.6. Intracellular Growth Assay

Ten isolated *Legionella* strains from different sources and different SBT types were selected and subjected to an intracellular growth assay repeated three independent times with *L. pneumophila* serogroup 1 strain (ATCC33152) as a positive control. All the strains were added to J774 cells, and the bacteria were stained using the Gimenez stain method after 0, 1, 2 and 3 days of infection and counted [23]. J774 cells were incubated in RPMI 1640 tissue culture medium including 10% calf serum at 37 °C with 5% CO_2_. Phosphate buffer saline (PBS) was used to dilute the bacteria to 1 × 10^8^ CFU/mL to obtain the primary dilution, which was then 10-fold diluted with culture medium containing J774 cells (2 × 10^5^ CFU/mL) to reach a multiplicity of infection (MOI) of approximately 10. Twenty-four-well dishes containing culture medium and bacteria were placed in the incubator at 37 °C, 5% CO_2_, and saturated humidity conditions. For the continuous culture, the culture medium was replaced by fresh medium each day. At days 0, 1, 2, and 3, we counted intracellular bacteria CFUs to determine growth. To measure internalization, PBS was used to wash away the extracellular bacteria and other potentially interfering substances. One milliliter of sterile, distilled water was added to the wells to release internal bacteria from the host cells, and the CFUs were determined by plating dilutions on BCYE agar plates (Oxoid Microbiological Products, Hampshire, UK).

### 2.7. Statistical Analysis

SPSS 19.0 for Windows (SPSS Inc., Chicago, IL, USA) was used to analyze the data. The chi square test or Fisher exact test were used for analyzing qualitative data, while the MANOVA of repeated measures or Mann-Whitney test were used to analyze quantitative data. The bacterial concentrations of *L. pneumophila* in the intracellular growth assay were analyzed by MANOVA of repeated measures. The differences were considered statistically significant when *p* < 0.05.

## 3. Results

### 3.1. Degree of Legionella Pollution in Water Samples

All 180 water samples were tested and among them 46 samples were *Legionella*-positive, from which we isolated 52 strains of *Legionella* (Table 2). Seventeen samples were *Legionella*-positive from 110 shower water samples with a positive rate of 15.5%. Fourteen samples from 60 hotel shower water samples were positive with a positive rate of 23.3% and 3 samples from 50 hospital shower water samples were positive with a positive rate of 6%. The positive rate of *Legionella* for the cooling tower water was at a relatively lower level of 13.3%. For 40 spring water samples, twenty-five samples were tested *Legionella*-positive giving the highest positive rate of 62.5%, which is significantly higher than the other two water samples from the shower and cooling tower (χ^2^ = 36.952, *p* < 0.01), there were no significant differences in the positive rate between the shower and cooling tower (χ^2^ = 0.083, *p* > 0.05). Overall, viable counts for the positive samples ranged from 20 CFU/100 mL to 10,720 CFU/100 mL. Of them, 33 water samples presented relatively lower counts, i.e., 20 CFU/100 mL to 100 CFU/100 mL. Viable counts from nine samples were between 100 to 1000 CFU/100 mL. Four samples exceeded 1000 CFU/100 mL with 1 of them reaching 10,720 CFU/100 mL. Notably, the samples with the counts over 100 CFU/100 mL were water from the hot springs.

### 3.2. Distribution of Serogroups of Legionella Isolates

In total, we isolated 52 strains from the 46 Legionella-positive water samples. Eight serogroup Legionella species were identified. Two distinct serogroups of Legionella species were simultaneously identified from six water samples. One sample from hotel shower and five samples from hot spring were detected *L. pneumophila* of serogroup LP2 and LP3.LP1 accounted for the most frequent serogroup for all three water sources (32.7%) with LP3 being a close second (28.9%) (Table 3).

### 3.3. Fluorescence Quantitative Polymerase Chain Reaction Analysis

The 5SrRNA gene was used to determine *Legionella* species as it was highly conserved among all of them. The *dot* gene located within the pathogenicity island of *L. pneumophila* was used to differentiate *L. pneumophila* and non-*L. pneumophila* bacteria [20]. Combing these two genes to analyze isolated strains, it was found that all the 52 strains were 5SrRNA gene- and *dot* gene-positive.

### 3.4. PFGE Analysis of Legionella Isolates

**Figure 1:**
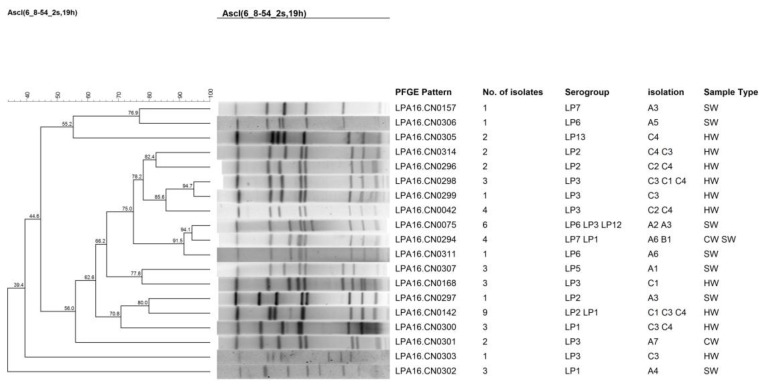
Clustering results of patterns obtained by pulsed-field gel electrophoresis (PFGE) analysis of the 52 *Legionella* strains isolated during 2015 to 2016. These strains of *L. pneumophila* were isolated from different water sources in Wenzhou from 2015 to 2016. A, hotels; B, hospitals; C, hot springs resorts; HW, hot springs water; CW, cooling tower water; SW, shower water. The number immediately after the letters under the “Isolation” column refers to the sampling site ID.

**Figure 2:**
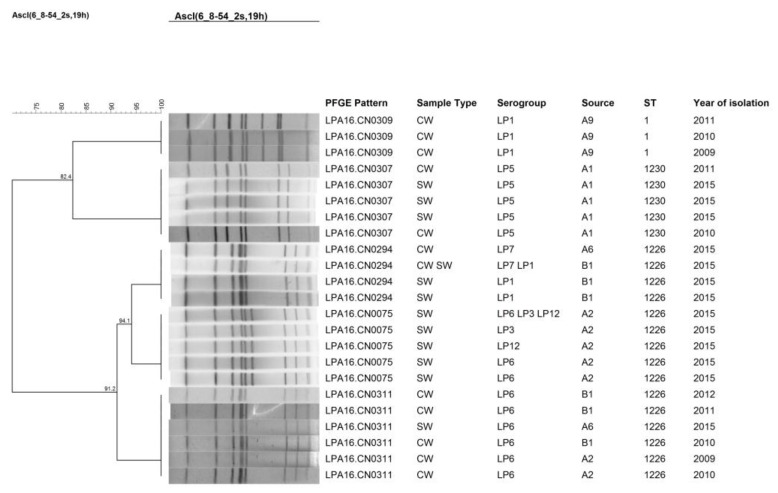
Clustering results of patterns obtained by PFGE analysis of strains chosen from samples of hotels and hospitals during 2009 to 2015. A, hotels; B, hospitals. The number immediately after the letters under the “Source” column refers to the sampling site ID.

The 52 isolates were further studied using PFGE where 19 different patterns were obtained (Figure 1). LPA16.CN0142 was the most frequently occurring pattern, which contained 9 strains that were isolated from water belonging to the hot springs. This was followed by LPA16.CN0075, which contained 6 isolates that came from shower water. LPA16.CN0142, LPA16.CN0075, and LPA16.CN0294 all corresponded with two or more kinds of serogroup isolates. Some different patterns of isolates were found from the same hot springs resort, while different hot springs resorts harbored isolates of the same patterns. To further study the genetic persistence of isolates from hotel and hospital water samples over time, isolates from 2009 to 2015 were examined by clustering analysis of PFGE patterns (Figure 2). The results revealed that some patterns of isolates of same sampling point persisted several years ago. The patterns of isolates from the hotel (A1) shower water in 2015 resembled patterns of isolates from the cooling tower water of the same hotel several years ago. A9 showed the persistence of the same PFEG pattern from the cooling tower water over time.

### 3.5. SBT Analysis of Legionella Isolates

During 2015–2016, fifty-two *L. pneumophila* isolates were differentiated by SBT into 18 different sequence types (STs) including ST7, ST9, ST87, ST114, ST1226, ST1230, and ST1469. Among these, the most frequent profile of STs was ST1226, isolated from shower water samples. The ST1226 corresponds to various serogroups, LP1, LP2, LP3, LP6, LP7, and LP12, to name a few. However, the serogroups have no direct correlations with the types of STs. 17 LP1-isolates were differentiated into seven types of STs, 15 LP3-isolates and 6 LP2-isolates were differentiated into six types of STs, while the rest of the isolates in this study had a single ST type. We isolated a total of 13 ST types with higher polymorphisms observed in the hot springs samples vs. those from other sources. Nine ST types (ST2202, ST2201, ST2203, ST2204, ST2197, ST2198, ST2199, ST2205, ST2196) were newly identified in this study and distinct from the types already in the EWGLI SBT database (Table 4).

**Figure 3:**
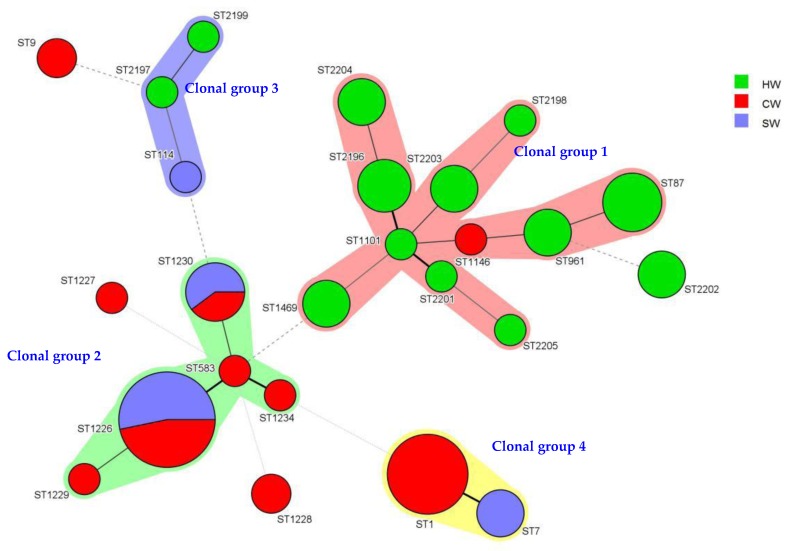
Minimum spanning tree analysis of 73 *Legionella* isolates from several water sources in Wenzhou, China. These strains were isolated from 2009 to 2016. In the minimum spanning tree, the sequence types (STs) are displayed as circles. The size of each circle indicates the number of isolates within this particular type with the STs are shown beside the circles. The colors of the halo surrounding the STs denote types that belong to the same clonal group. CW: cooling tower; HW: hot springs; SW: shower water.

To study the genetic relationship of the strains of *L. pneumophila* from different districts in Wenzhou beginning 2009, we conducted clustering analysis using BioNumerics. Seventy-three strains with 25 ST types were differentiated into four clonal groups and four singletons. Clonal group 1 contained 11 types of STs. Of these 11, 10 types were isolated from the hot springs. Clonal group 2 contained five ST types, all from the shower and cooling tower. Clonal group 3 contained three ST types, from the hot springs and shower. For clonal group 4, this group only had two types of STs, ST1 and ST7 from the cooling tower and shower. Interestingly, the cooling tower water isolates were mainly ST1 before 2015, and all the isolates’ serogroup was LP1 (Figure 3) (Table 4.).

### 3.6. Intracellular Growth Ability

**Figure 4:**
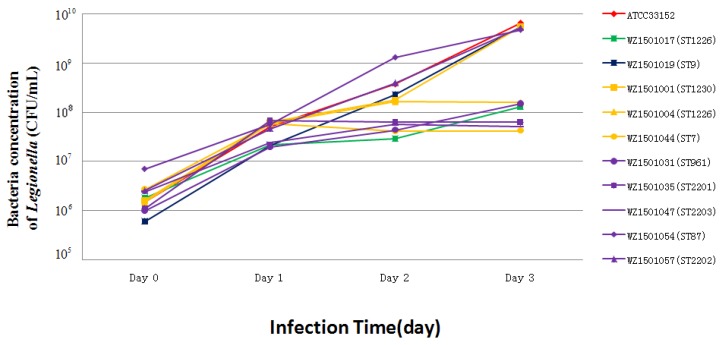
Intracellular growth of *Legionella* isolates within J774 cells. WZ1501001 (SW-H2), WZ1501004 (SW-H2), WZ1501017 (SW-H1), WZ1501019 (CW), WZ1501031 (HW), WZ1501035 (HW), WZ1501044 (SW-H2), WZ1501047 (HW), WZ1501054 (HW), WZ1501057 (HW). CW: cooling tower, blue line; HW: hot springs, purple line; SW-H1: hospital shower, green line; SW-H2: hotel shower, yellow line; Control, ATCC33152, red line.

Mouse BALB/c crophage cell line J774 was used for testing the intracellular growth ability of *Legionella* isolates as *L. pneumophila* serogroup 1 (ATCC33152 was used as a positive control) (Figure 4). Gimenez staining on day 2 of J774 cell infection allowed observation under a light microscope, which showed that all the ten isolates had intracellular growth ability in J774 cells. To further evaluate the ability of the strains, bacteria numbers were counted. Interestingly, six strains (group 1) exhibited continuous growth within 72 h under the standard assay’s condition and four strains (group 2) showed significant growth within 48 h after which they plateaued at 72 h (Figure 4). The group 1 strains consisted of 6 strains: ST1230, ST1226, ST9, ST961, ST87, and ST2202, which came from the cooling tower, showers, and hot springs. The strains of group 2: ST1226, ST2201, ST7 and ST2203 belonged to those from the showers and hot springs. Although the sources of the two groups overlap, the intracellular growth ability of the strains is shown to be significantly different (*p* < 0.05) (Figure 4). Strains from the same source may exhibit different intracellular growth abilities; the WZ1501001 (ST1230) strain showed a higher growth ability than WZ1501044 (ST7) although these two strains were isolated from the same source, i.e., hospital shower. On the other hand, strains of the same ST type may also show different intracellular growth abilities. The growth ability of WZ1501004 (ST1226) from group 1 was significantly higher than WZ1501017 (ST1226) from group 2. Taken together, the results show that intracellular growth abilities are not correlated with their sources or ST types.

## 4. Discussion

This study suggests a high prevalence of *Legionella* in Wenzhou’s hot springs. *Legionella* was detected in more than 60% of collected hot springs water samples both in summer and winter, and the serogroups of LP1 and LP3 mostly accounted for the group of detected *Legionella* spp., which in fact are the two main serogroups causing Legionnaires’ disease. The viable count assay revealed the high abundance of *Legionella* in some hot springs. The concentration of *Legionella* was observed to be over 1000 CFU/100 mL in 4 samples. Among them, 1 sample had up to 10,720 CFU/100 mL. When above normal levels or an increase in *Legionella* spp. number greater than 100 CFU/100 mL of water is calculated, policies should be enforced to eradicate the bacteria from the contaminated water source [24]. To date, the infective dose has not yet been precisely determined. Estimated data indicates that in the case of water contamination by *Legionella*, 10^4^ to 10^6^ CFU/100 mL may lead to the disease occurring sporadically, but when the counts exceed 10^6^ CFU/100 mL, a Legionnaire’s disease outbreak can be expected [25,26,27]. In this study, although only one hot spring sample had enough *L. pneumophila* (10,000 CFU/100 mL) to potentially cause sporadic disease, three other hot spring samples had elevated amounts of the bacteria. Although the 2011 tourism industry standard of China, Classification and accreditation for service-rated hot springs enterprise, claims that *L. pneumophila* has not been detected in the hot springs, the detection range was not being defined and the standard detective method was not established, indicating that the industry standard for detection of *L. pneumophila* in China should be improved.

Shower water may carry pathogens that come into contact with people, thus is directly related to a person’s health. The *Legionella*-positive rate of hotel and hospital showers was 15.5%. Currently, the cleaning regulations and monitoring standards for *L. pneumophila* in central air conditioning of public areas are made available, however, disinfection and detection standards for shower systems have not been set up. The concentration of the bacteria in *Legionella-*positive shower samples ranged from 20 to 100 CFU/100 mL. Shower water originates from hot water shower tanks and are piped to each room’s showers, therefore, once the bacteria colonizes in tanks or pipes, it would spread to every part of the building. All the isolated strains from the hospital showers were serogroup LP1; the situation was very different from that of hotels where the most common serogroups were LP1, LP5, and LP6.Cancer patients, postoperative patients, and patients under long-term immunosuppressive treatment that are more susceptible to infection can be at risk once *L. pneumophila* invades the hot water system.

The PFGE results revealed that the patterns of isolates from different rooms with tin the same collection point were not different, suggesting that they share the same pipe system. It was observed that some *L. pneumophila* strain patterns were detected in certain hospitals’ and hotels’ showers in different years, indicating that the same strain patterns may be persisting for a long time in those particular water sources. However, the patterns of isolates from different hot springs resorts varied widely, with some samples from the same collection point exhibiting several different strain patterns.

SBT results showed that the population of STs was highly diverse in the Wenzhou area. Fifty-two isolates from the year 2015 to 2016 were divided into 18 STs, including ST7, ST9, ST87, ST114, ST1226, ST1230, and ST1469. Isolates from 2009 to 2016 were divided into 25 STs, 4 clonal groups and 4 singletons. ST1226 was the most common type found in local hot springs and also existed in the cooling tower over the years. ST1 was the most dominant ST-type of *L. pneumophila* in the cooling tower from 2009 to 2014 and this result was consistent with ST-type distribution of *L. pneumophila* in the cooling tower in Shijiazhuang (China) [28,29]. However, from 2015 to 2016, the ST1 type was not detected, but ST7, which differs only in one allele from ST1, was observed instead.

According to the SBT database, the strains of ST87, ST7, ST114, ST9, and ST961 isolated in our study previously caused Legionnaires’ diseases in some countries. ST87 was detected in all three hot spring resorts and their serogroups were all LP3, which agrees with the strain noted in the SBT database and was also reported to be present in a hot springs resort in Beijing [23]. 188 strains of ST9 were available in the SBT database (last accessed: 18 May 2016), with 125 of them isolated from clinic samples, which corresponds with one ST9 strain we isolated from the cooling tower in this study. ST7 and ST114 were detected in shower water samples with the serogroups LP1 and LP6, respectively, of which the results are consistent with the available strains in the SBT database. Nine strains of ST type (from ST2196 to ST2205) specific to the Wenzhou area were first detected in this study; of them, 7 strains including ST2196 and ST87 belong to one clonal cluster. However, the newly isolated ST type ST2197, is in the same clonal cluster belonging to ST2199 and ST114.

It was found that the serogroup and ST type of *L. pneumophila* from the hotels’ and hospitals’ showers were almost unchanged with that of the same sampling points of recent years. Although the PFGE types of strains from two hotels varied within the recent years, the similarities within these strains were up to 91.2%. The results may be due to the higher resolution of PFGE for typing bacteria groups vs. the capacity of multilocus sequence typing (MLST). Some mutations may have occurred during the bacteria’s generational propagation and caused the PFGE variations that were observed, but may not be detected by MLST. Thus, PFGE and SBT are complementary approaches for group differentiation of *L. pneumophila*.

The pathogenic mechanism of *L. pneumophila* is that the bacteria has the ability to invade into macrophages of the host’s lung and reproduce until the cells collapse and release cellular contents to ultimately initiate cascades of immune reactions leading to lung damage. Thus, the intracellular growth ability assay could be an effective way for measuring the pathogenicity of *L. pneumophila* [30]. We studied the intracellular growth ability of isolates from artificial water sources for investigating their pathogenicity. All the isolates from all the various water sources: hot springs, cooling tower, and showers revealed that they could infect mouse J774 macrophages, indicating a possible high risk for people living in the Wenzhou area to contract Legionnaires’ disease.

## 5. Conclusions

Our study demonstrates the high prevalence of *Legionella* in the hotel and hospital showers, cooling towers, and hot springs in Wenzhou, China. Furthermore, the SBT and PFGE methods showed high genetic polymorphisms of *Legionella*; the same types of strains have persisted in certain water sources, although some PFGE and SBT type variations were found. Moreover, testing for the intracellular growth abilities of the isolates proved that they are pathogenic. Due to the possibility of a public outbreak of *Legionella* infection stemming from the colonization of the showers, cooling towers, and hot springs, improved disinfection and prevention strategies are critical and urgently needed.

## Figures and Tables

**Table 1:** Oligonucleotides used for real-time amplification of *L. pneumophila*.

Name	Positions on Gene	Sequence (5’→3’)	Fragment Size (bp)	GenBank Accession Number of Reference Sequence
5SF	618682–618701	ACTATAGCGATTTGGAACCA	104	CP01760.1
5SR	618785–618766	GCGATGACCTACTTTCGCAT		CP01760.1
5SProbe	618737–618759	HEX-CCGCGCCAATGATAGTGTGAGGC-BHQ		CP01760.1
dotAF	986–1004	ATTGTCTCGCGCGATTGC	81	AF095231
dotAR	1066–1043	CCGGATCATTATTAACCATCACC		AF095231
dotA probe	1006–1027	FAM-ATACAGCAAATGTATGTGACTT-MGB		AF095231

**Table 2:** *Legionella*-positive rates from different water sources in Wenzhou from 2015 to 2016.

Water Type	No. of Tested Samples		No. of Positive Samples	Positive Amounts
SW-H1	110	50	3	15.45% (17/110)
SW-H2		60	14	
CW-H1	30	10	0	13.33% (4/30)
CW-H2		20	4	
HW	40		25	62.50% (25/40)
Total	180		46	25.56% (46/180)

CW: cooling tower; HW: hot springs; SW-H1: hospital shower; SW-H2: hotel shower.

**Table 3:** Distribution and serogroup characteristics of the 52 strains of *L. pneumophila* isolated from water samples of various sources during 2015 to 2016 in Wenzhou, China.

Serogroup	Hotel Shower Water (n = 15)		Hospital Shower Water (n = 3)		Hot Springs Water (n = 30)		Cooling Tower Water (n = 4)		Total (n = 52)	
	No.	Proportion (%)	No.	Proportion (%)	No.	Proportion (%)	No.	Proportion (%)	No.	Proportion (%)
LP1	3	20.00	3	100	11	36.67	0	0.00	17	32.69
LP2	1	6.67	0	0.00	5	16.67	0	0.00	6	11.54
LP3	1	6.67	0	0.00	12	40.00	2	50.00	15	28.85
LP5	3	20.00	0	0.00	0	0.00	0	0.00	3	5.77
LP6	5	33.33	0	0.00	0	0.00	1	25.00	6	11.54
LP7	1	6.67	0	0.00	0	0.00	1	25.00	2	3.85
LP12	1	6.67	0	0.00	0	0.00	0	0.00	1	1.92
LP13	0	0.00	0	0.00	2	6.67	0	0.00	2	3.85

**Table 4:** Profiles of sequence types (STs) from different sources during 2009 to 2016 in Wenzhou.

Year	Source	ST	Serogroup	FlaA	PilE	Asd	Mip	Momps	ProA	NeuA	No. of Isolates
2015–2016	HW	1101	LP1	6	6	15	3	9	14	11	1
		2202	LP1	2	10	17	14	21	14	8	3
		2201	LP1	17	6	15	3	9	14	11	1
		2203	LP1	6	6	15	3	9	4	3	3
		2204	LP1	6	6	15	3	4	4	13	3
		2197	LP2	3	5	1	7	14	11	8	1
		2198	LP2	6	10	15	7	17	4	3	1
		2199	LP2	7	5	1	7	14	32	8	1
		2205	LP2	17	10	15	7	17	14	11	1
		87	LP3	2	10	3	28	9	4	13	5
		961	LP3	2	10	3	28	9	14	11	3
		1469	LP3	2	6	17	3	9	11	11	3
		2196	LP2, LP3, LP13	6	6	15	3	4	14	11	4
	CW	9	LP3	3	10	1	3	14	9	11	2
		1226	LP7, LP6	7	10	17	28	13	11	3	2
	SW-H1	1226	LP1	7	10	17	28	13	11	3	3
	SW-H2	7	LP1	1	4	3	1	1	1	6	3
		1230	LP5	7	6	17	6	13	11	40	3
		114	LP6	3	6	1	6	14	11	9	1
		1226	LP2, LP3, LP6, LP7, LP12	7	10	17	28	13	11	3	8
2009–2014	CW	ST1226	LP6	7	10	17	28	13	11	3	5
		ST1234	LP6	1	6	17	28	13	11	3	1
		ST1227	LP7	11	14	16	19	15	13	3	1
		ST1228	Lp12, LP5	8	6	34	9	2	8	3	2
		ST1229	LP6	9	10	17	6	13	11	3	1
		ST1146	LP1	6	10	15	28	9	14	11	1
		ST583	LP6	7	6	17	28	13	11	3	1
		ST1230	LP5	7	6	17	6	13	11	40	2
		ST1	LP1	1	4	3	1	1	1	1	7

CW: cooling tower; HW: hot springs; SW-H1: hospital shower; SW-H2: hotel shower.
